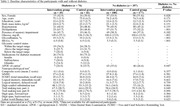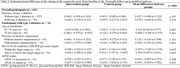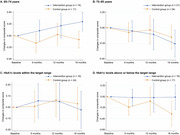# Multimodal intervention for risk reduction of dementia in older adults with type 2 diabetes: Post‐hoc sub‐group analyses of the J‐MINT

**DOI:** 10.1002/alz.088032

**Published:** 2025-01-09

**Authors:** Taiki Sugimoto, Kazuaki Uchida, Nanae Matsumoto, Kosuke Fujita, Yoko Yokoyama, Yujiro Kuroda, Takuya Omura, Akinori Nakamura, Paul K. Crane, Hidenori Arai, Takashi Sakurai, J‐MINT study group

**Affiliations:** ^1^ National Center for Geriatrics and Gerontology, Obu, Aichi Japan; ^2^ University of Washington, Seattle, WA USA

## Abstract

**Background:**

This post‐hoc sub‐group analysis of the Japan‐multimodal intervention trial for prevention of dementia (J‐MINT) aimed to examine the efficacy of the multi‐domain intervention in older adults with type 2 diabetes.

**Method:**

J‐MINT was an 18‐month, randomized controlled trial. Participants aged 65‐85 years with mild cognitive deficits were recruited and randomized into multidomain intervention (management of vascular risk factors, physical exercise, nutritional counseling, and cognitive training) and control groups (written health‐related information every 2 months). The outcome was the change from baseline to 18‐month follow‐up in a composite score derived from several neuropsychological tests, including tests of global cognitive function, memory, attention, and executive function/processing speed. Among individuals with diabetes, stratified analyses based on age at enrollment (65‐74 years vs. 75‐85 years), presence of memory impairment (with vs. without memory impairment), HbA1c levels according to the Japan Diabetes Society/Japan Geriatric Society recommended target range (within vs. above/below the target range), and *APOE* phenotype (0 vs. ≥1 *APOE* ε4 alleles), were performed using mixed models for repeated measures.

**Result:**

Out of 531 participants enrolled in J‐MINT, 76 participants with type 2 diabetes and 356 without had at least one post‐baseline assessment and were included in the analysis. In participants with and without diabetes, there were no significant effects of the intervention on changes in the composite score. Among people with diabetes, stratified analyses revealed that the intervention effect was significant in individuals aged 65‐74 years (mean difference between the intervention and control groups, 0.310 [95% CI 0.104–0.517], *p* = 0.003). Moreover, the intervention appeared to be effective in those with HbA1c levels above/below the target range (0.304 [0.007–0.601], *p* = 0.045), although it did not reach statistical significance after Bonferroni correction (*p* < 0.005). There was no significant effect of intervention in stratified analyses based on memory impairment and the *APOE* phenotype.

**Conclusion:**

These results suggest that multidomain interventions may be particularly valuable for people with diabetes who are in the younger age group or whose glucose values are not in the target range. However, this should be confirmed in other data sets.